# Intestinal Atresia: Experience at a Busy Center of North-West India

**DOI:** 10.21699/jns.v5i4.405

**Published:** 2016-10-10

**Authors:** Shilpi Gupta, Rahul Gupta, Soumyodhriti Ghosh, Arun Kumar Gupta, Arvind Shukla, Vinita Chaturvedi, Praveen Mathur

**Affiliations:** Department of Paediatric Surgery, SMS Medical College, Jaipur, Rajasthan, India

**Keywords:** Intestinal atresia, Neonatal, Intestinal obstruction

## Abstract

**Objective:** To evaluate the presentation, management, complications and outcome of intestinal atresia (IA) managed at our center over a period of 1 year.

**Materials and methods:** Records of patients of IA admitted in our center from January 2015 to December 2015 were retrospectively analyzed. Demographic data, antenatal history, presenting complaints, location (duodenal, jejunoileal, colonic) of atresia, surgery performed and peri-operative complications were noted.

**Results:** Total 78 cases of IA were included in the analyses. Mean age and weight at the time of presentation was 5.8 days (range 0-50), and 1.9 kg (range 1.1-3.2), respectively. IA included duodenal atresia [DA (32)], jejuno-ileal atresia [JIA (40)], colonic atresia [CA (3)] and atresia at multiple-location (sites) in 3 cases. Ninety percent of patients underwent surgery within 5 to 20 hours of admission. All cases of DA except one underwent Kimura's diamond shaped duodeno-duodenostomy. One case with perforated duodenal web underwent duodenotomy with excision of web. Seven patients with JIA and CA required primary stoma, while rest were managed by excision of dilated proximal segment and primary anastomosis. Complications included anastomotic leak in 5, proximal perforation in 2, functional obstruction in 7, aspiration pneumonitis in 3, and wound infection in 6 patients. Mean hospital stay for survivors was 11 days. Overall survival was 63%.

**Conclusion:** Late presentation, overcrowding in intensive care unit, septicemia, functional obstruction and anastomotic leak are the causes of poor outcome in our series. Early diagnosis, some modification in surgical technique, use of total parenteral nutrition and adequate investigations for other congenital anomalies may improve the outcome.

## INTRODUCTION

Intestinal atresia (IA) is one of the most common causes of intestinal obstruction in neonates [1]. Reported survival rates in most of the series from developed countries are 90% or more [2-4]. This has been achieved because of refinements in neonatal intensive care, operative technique, use of total parenteral nutrition (TPN), antenatal diagnosis and neonatal anesthesia [5]. However, survival rates in developing countries are still lower (58.3-71.5%) [4-8]. In two reports from India, survival rate of 82% and 80% has been reported in series of 46 patients of jejuno-ileal atresia (JIA) and colonic atresia (CA), and 15 patients of duodenal atresia (DA), respectively [9,10].

## MATERIALS AND METHODS

Records of all patients of neonatal IA treated at our center during one-year period (January 2015 to December 2015) were analyzed. All patients were admitted in neonatal surgical intensive care unit (ICU). Data of demographics, antenatal history, presentation, location and type of IA (duodenal, jejuno-ileal, colonic), and peri-operative complications were collected. Patients of IA with volvulus, complicated meconium ileus and gastroschisis were excluded from our series.

After the presumptive diagnosis was made on the basis of clinical assessment, an upright X-ray abdomen was taken. Decision of surgery was taken on the basis of clinical and radiological assessment. Nasogastric (NG) tube was inserted in all patients. Intravenous fluids and antibiotics were started and continued post-operatively. NG tube was removed after 5th post-operative day when NG aspirate was gastric and less than 15ml/day, and thereafter gradual feeding was started. Follow up was not included in the study.

## RESULTS

Total 78 patients (45 male, 33 females) met the inclusion criteria. Mean age and body-weight on presentation were 5.8 days (range 0-50) and 1.9 kg (range 1-3), respectively. Twenty percent of patients presented at age of more than one week. IA included DA (n=32), JIA (n=40), CA (n=3) and atresia at multiple-location (sites) in 3 cases. Two of the 32 cases of DA had associated esophageal atresia (EA) with tracheo-esophageal fistula (TEF), and anorectal malformation (ARM) labeled as triple atresia. Antenatal history of polyhydramnios could be elicited in 6 cases, and only one neonate with DA was antenatally diagnosed.

Vomiting was the chief presenting complaint in DA, while abdominal distension, bilious vomiting, and failure to pass meconium, were the presenting symptoms in JIA and CA. Double-bubble sign on upright X-ray abdomen was present in cases of DA; while distal gas with double-bubble was seen in patients with perforated duodenal web (Fig.1A). Fig.1B shows double-bubble sign with red rubber catheter in upper esophageal pouch in a case of triple atresia. One case of DA had associated gastric perforation (Fig.1C). Upper-GI gastrografin contrast study was performed in 4 cases of perforated duodenal web because of diagnostic dilemma due to presence of gas in distal bowel on X-ray. Tripple-bubble sign, and varying degree of dilated bowel loops and multiple air-fluid levels were seen in JIA (Fig.2A,B,C). Gastrografin enema was performed in two cases of ileal atresia who presented more than 15 days after the birth. Both of them showed unused colon (Fig.2D,E).

Majority of cases (90%) were operated within 5 to 20 hours of admission. Table 1 shows type of operative procedures performed for different IAs, and their outcomes in terms of survival. Total 31 cases of DA were operated. One patient with DA expired before intervention. Intra-operative findings include type I atresia in 13 (42%), Type II in 1 (3.2%), and type III in 17 (54.8%). Among type I DA cases, perforated web was found in 3, and annular pancreas in 5 cases. Operative findings of JIA included type I in 8 (20%), type II in 3 (7.5%), type IIIa in 18 (45%), type IIIb in 3 (7.5%) and type IV (multiple JIA) in 8 (20%). Fig.3 shows intraoperative findings in cases of DA and JIA at different locations. CA was present in 3 cases. Two were type I, and 1 had type III CA. A rare association of gallbladder duplication with IA was found in two cases; one with type III DA and other with multiple-site (pyloric, ileal, colonic) IA.

All the operated cases of DA were managed by Kimura’s diamond shaped duodeno-duodenostomy except one in which duodenotomy and excision of perforated web was done. One case had associated gastric perforation which was repaired. One patient with triple atresia died before surgery due to severe pneumonitis. The other case of triple atresia was subjected to primary repair of EA, Kimura’s duodeno-duodenostomy and colostomy.

In JIAs, dilated proximal bowel (length ranged from 2 to 20 cm) was resected and distal patency was checked. Bowel continuity was maintained by end to oblique single layer anastomosis. In 8 cases of type IV JIA (multiple JIA), the whole segment of bowel containing atresias was resected. In 4 of these 8 cases, single anastomosis was performed following bowel resection, while 2 required anastomosis at two different sites to avoid severe shortening of bowel length, and 2 cases of jejunal and distal ileal atresia underwent jejuno-jejunal anastomosis with ileostomy. Three case of CA underwent resection of atretic segment followed by colo-colic anastomosis in one, colostomy in one and covering ileostomy in one patient. Details of 3 patients with multiple-site atresia have been given in Table 1. Among total 77 operated patients, stoma was created primarily in 7 patients (ileostomy in 6 and colostomy in 1). Six patients needed re-exploration and stoma was created in 3 of these.

Post-operative complications, their management and outcome are shown in Table 2. Anastomotic leak occurred in 5 patients (all JIAs). Three of them were re-operated, stoma was created in two at the site of anastomotic leak and one was re-anastomosed. Two patients with anastomotic leak died of septicemia before intervention. Two patients with DA and five with JIA had prolonged bile drainage of > 30 ml/day from NG tube for more than 10 days, due to functional intestinal obstruction. Upper-GI contrast study of these patients showed anastomotic patency but persistent dilated loops and air-fluid levels proximal to anastomotic site. One of them died due to aspiration pneumonitis, and two due to septicemia. One patient underwent re-exploration, and anastomosis was found patent, with proximal dilated bowel. Resection of 8 cm of dilated bowel and re-anastomosis was done. Remaining three patients were managed conservatively. Proximal perforation occurred in two. Repair of perforation in one, and repair of perforation with ileostomy at the site of previous anastomosis in the other patient was performed. Wound infection occurred in 6 patients, 5 of them managed with dressings, one needed secondary suturing.

Mean hospital stay for survivors with DA was 11 days (range 8-24) and for JIA and CA was 11 days (range 5-27). In our series survival rate was 61% for DA, 65% for JIA and 66.6% for both colonic and multiple-site atresia. Overall survival was 63%.

**Figure:**
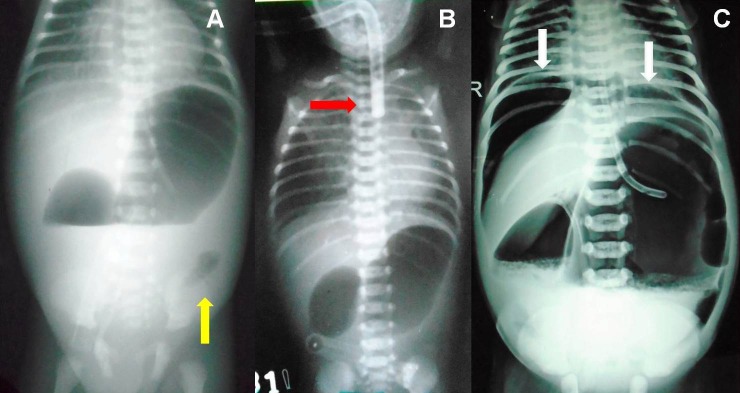
Figure1: Double-bubble appearance with distal gas in a case of perforated duodenal web (A); double-bubble sign with red rubber catheter in upper esophageal pouch in a case of triple atresia (B); Double-bubble sign with pneumoperitoneum in a case of duodenal atresia with gastric perforation (C).

**Figure:**
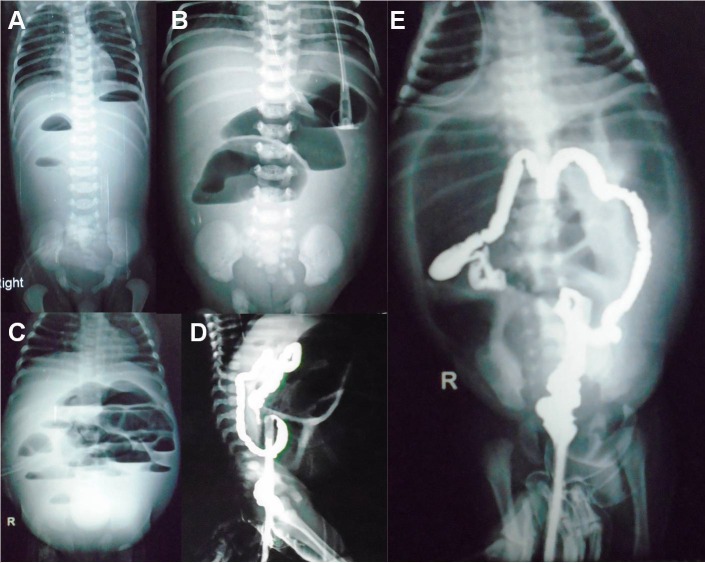
Figure 2: Triple-bubble sign (A) in a case of jejunal atresia; dilated bowel loops with a few air-fluid levels in a case of jejunal atresia (B); dilated bowel loops with multiple air-fluid levels a case of ileal atresia (C); gastrografin enema showing unused colon in a case of ileal atresia (D,E).

**Figure:**
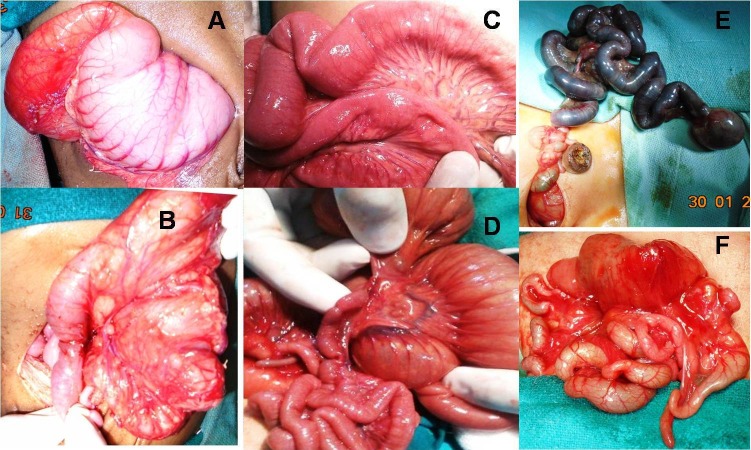
Figure 3: Intra-operative images showing dilated stomach and proximal duodenum (A), with perforated duodenal web (B); ileal web (C); jejunal atresia type II (D), jejuno-ileal atresia type IIIb (E); type IV jejuno-ileal atresia (F).

**Figure:**
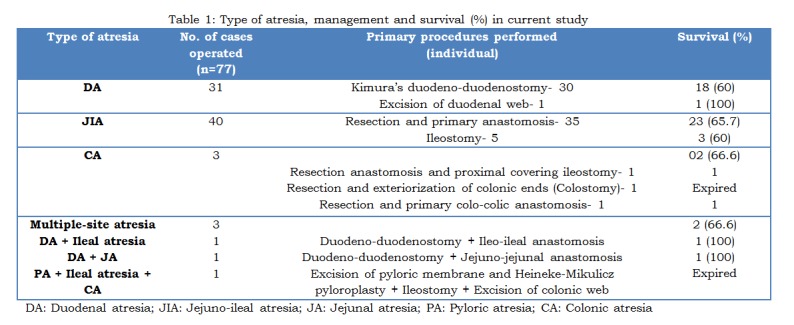
Table 1: Type of atresia, management and survival (%) in current study

**Figure:**
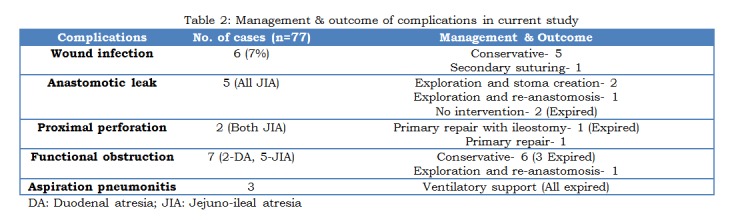
Table 2: Management and outcome of complications in current study

## DISCUSSION

IA is one of the most common causes of neonatal intestinal obstruction. Regarding etiology of IA, two theories are Tandler’s concept of a lack of re-vacuolization of solid cord stage of intestinal development [9] and Louw and Barnard theory of late intrauterine mesenteric vascular accident [10]. Prenatal ultrasonography is more reliable in detection of duodenal atresia than JIA or CA. Basu and Burge reported that 31% of patients with small bowel atresia could be diagnosed on prenatal ultrasound [11]. Pre-operative management includes, primary resuscitation, correction of dehydration and electrolyte abnormalities. Because of high incidence of cardiac and renal anomalies associated with DA, echocardiography and ultrasonography of the abdomen should be performed [12]. In a series of 138 cases of DA, 38% cardiac anomalies, 14% renal anomalies, 6% EA with TEF, and 5% imperforated anus were noted [5]. In current series EA with TEF and imperforate anus was found in 2 (6%) patients of DA, and no investigations were performed for detection of cardiac and renal anomalies. Two cases of gallbladder duplication, one with DA and other with combined pyloric, ileal and colonic atresia, were noted in current series. Gallbladder duplication with IA is an extremely rare association. Only three such cases have been reported in literature [13]. Two of these cases are the part of current series.

Surgical procedure should be individualized according to location and type of atresia, degree of dilatation of proximal segment and pre-operative condition of the patient. Most common procedure performed for DA is Kimura’s diamond shaped duodeno-duodenostomy. In type I DA, duodenotomy with excision of web is an option which is performed less commonly due to chances of damage to ampulla of Vater. Intra-operatively compression of gallbladder with release of bile may help in identification of ampulla [5]. Duodeno-jejunostomy is another option in difficult cases, because of patient anatomy particularly in small and premature children. In current series only one case was managed with duodenotomy and web excision. Anti-mesenteric tapering duodenoplasty is advised as a useful technique in managing duodenal motility disorder related to mega-duodenum [14].

Surgical procedure for JIA should be based on site and type of atresia, proximal segment dilatation, length of remaining bowel and pre-operative general condition of patient. To avoid functional obstruction and abnormal motility in retained proximal bowel, dilated segment can be excised up to ligament of Treitz. Some reports suggest that resection of dilated proximal bowel should be minimized, as TPN can take care of the period of dysmotility and impaired anastomotic function[15]. However preservation of as much bowel length as possible at the risk of creating a poorly functioning anastomosis can produce significant morbidity and mortality [16,17]. In instances of short bowel length, proximal tapering enteroplasty or intestinal plication has been proposed as an alternative, in an effort to preserve bowel length [18-21]. In current series, limited (2 to 20 cm) resection of proximal bowel was done. Tapering enteroplasty was not done in any case. Stoma should be avoided in patient with IA because it increases the morbidity and mortality. Indications to create stoma in our series were either presence of sepsis and severe peritonitis, poor general condition or gross discrepancy between the caliber of proximal and distal bowel segments in cases of distal ileal and colonic atresia.

In current study peri-operative mortality is almost same for DA and JIA. Outcome of IA is still not satisfactory in developing countries [6,7,22]. Difference in outcome in developed and developing countries is not because of surgical techniques, rather because of advancement in the following: 1) availability of good primary health care with early referral and proper transportation system, 2) availability of neonatal surgeons, 3) parallel growth of neonatal anesthesia, 4) development and availability of sophisticated medical devices, 5) availability of neonatal ventilators and respiratory support system, 6) TPN, 7) neonatal ICU with trained personnel [23].

In developing countries, delay in diagnosis, late presentation to tertiary center, poor primary health care infrastructure and transportation system, lack of equipped neonatal ICU with trained personnel, overcrowding leading to cross infection and septicemia, and unavailability of TPN, are the primary reasons for high mortality rates.

Early diagnosis reduces pre-operative metabolic derangements and facilitates appropriate and rapid surgical treatment, which positively impacts the rate of survival [24,25]. Pre-operative resuscitation, adequate correction of hypovolemia, third space loss and electrolyte imbalance in neonates undergoing major GI surgery is invaluable for the outcome of surgery [26]. Cardiac anomalies are more commonly associated with DA than JIA and are one of the major obstacles to successful outcome in case of DA [5].

## CONCLUSION

Survival of neonates with IA is still poor in such a high volume tertiary care center in India (developing country). Poorer outcome in current series may be due to delayed presentation, high patients load, overcrowding in the ICU and septicemia. Functional obstruction and anastomotic leaks are the major surgical complications. Some modifications in surgical techniques such as tapering enteroplasty, use of TPN, and adequate investigations for congenital cardiac anomalies, may improve the outcome.

## Footnotes

**Source of Support:** None

**Conflict of Interest:** None

**Note: **First two authors contributed equally in the manuscript
